# The Microcirculation in Sepsis

**Published:** 2009-06

**Authors:** Asha Tyagi, Ashok Kumar Sethi, Gautam Girotra, Medha Mohta

**Affiliations:** 1 Reader, Department of Anaesthesiology & Critical Care, University College of Medical Sciences & Guru Teg Bahadur Hospital, Shahadra, Delhi-110095, India; 2 Professor & Head, Reader, Department of Anaesthesiology & Critical Care, University College of Medical Sciences & Guru Teg Bahadur Hospital, Shahadra, Delhi-110095, India; 3 Senior Resident, Department of Anaesthesiology & Critical Care, University College of Medical Sciences & Guru Teg Bahadur Hospital, Shahadra, Delhi-110095, India; 4 Reader, Department of Anaesthesiology & Critical Care, University College of Medical Sciences & Guru Teg Bahadur Hospital, Shahadra, Delhi-110095, India

**Keywords:** Microcirculation, Sepsis, Micro-haemodynamics

## Abstract

**Summary:**

Sepsis is a leading cause of mortality in critically ill patients. The pathophysiology of sepsis involves a highly complex and integrated response, including the activation of various cell types, inflammatory mediators, and the haemostatic system. Recent evidence suggests an emerging role of the microcirculation in sepsis, necessitating a shift in our locus away Irom the macrohaemodynamics to ill icrohaemodynanmics in a septic patient. This review article provides a brief overview of the microcirculation, its assessment techniques, and specific therapies to resuscitate the microhaemodynamics.

## Introduction

Sepsis and its progression to severe sepsis, septic shock and multiple organ dysfunction syndrome is a major cause of ICU admissions and mortality^1^. Severe sepsis and septic shock may be characterized by a derangement in global cardiac indices typically leading to low peripheral resistance, which the body tries to compensate for by increasing the cardiac output. However, despite this increase in cardiac output, the tissues are unable to utilize oxygen as evidenced by the high lactate levels, deranged acid-base balance, and increased gastric carbondioxide level. The presence of tissue hypoxia despite adequate systemic oxygen transport has been blamed on altered microhaemodynamics as well as in itochondrial dysfunction during sepsis^2^. However, the relative contributions of disturbed microcirculation and impaired mitochondrial function for sepsis related tissue dysoxia are still debatable. The present review aims to highlight the former cause of tissue hypoxia in sepsis i.e., involvement of the microcirculation. It moves from recapitulating relevant anatomy of microcirculation, to its current role in pathophysiology of sepsis, optimization during sepsis and lastly the modalities for its assessment.

## Functional anatomy of microcirculation

Anatomically, the microcirculation consists of the arterioles, terminal arterioles, capillaries, and post-capillary and collecting venules^3^. Rather than dwell on different vessels of the microcirculation as per their anatomical designations, it is clinically more relevant to divide the microvascular bed functionally into resistance, exchange and capacitance vessels.

*Resistance vessels (Arterioles)*. Arteriole (<100-200 μm in diameter) is the final branch of the arterial system and marks the beginning of microcirculation. The arteriole and proximal part of its successor - terminal arteriole, are heavily infested with smooth muscle cells making them the mainstay of controlling the resistance in the microcirculation, by having the ability to change their caliber due to presence of smooth muscle cells. Terminal arteriole is the last division of arteriolar network and terminates into a capillary network without anastomosis with any other arterial or venous vessel.

*Exchange vessels (Capillaries)*. Capillaries are essentially tubes lined by a single layer of endothelium, containing no smooth muscle cells and thus being unable to change their diameter actively. The structure is specialized to maintain their primary function as exchange vessels. Endothelium lining the capillaries varies from being non-fenestrated to fenestrated or discontinuous, in different organs according to their metabolic requirements. There are over 10 billion capillaries (5-9 μm) in the body. Capillary density refers to the number of capillaries present in a given area or volume of tissue. The body can cope up with increased metabolic demands by “capillary recruitment” i.e., increasing the proportion or perfused capillaries. The intrinsic ability for capillary recruitment also serves to decrease the total resistance, since capillary vessels are arranged in parallel rather than in series. This latter advantage of capillary recruitment is however offset by the rather small contribution of capillaries to resistance as compared to that offered by the arterioles. The more beneficial effect of capillary recruitment is the increase in the exchange-vessel surface area exposed to flowing blood, enabling significant increase in the exchange of metabolites and gases. Recruitment primarily occurs by opening whole bundles or capillaries, while the perfusion of connections between already open vessels only plays a minor role. When two capillaries converge, a post-capillary venule is formed. Though slightly larger than a capillary (15-20 μm) it also lacks smooth muscles, and is unable to regulate its caliber. Most of the exchange of fluid, nutrients and end products occurs in this part of circulation and hence capillaries and non-muscular venules are termed as exchange vessels.

*Capacitance vessels (Venules)*. Venules with diameter greater than 30 μm start acquiring smooth muscles cells. These muscular venules and veins are termed ‘capacitance vessels’ since they hold almost 70% of the total circulating blood volume while having negligible contribution to resistance.

**Characteristics of microcirculation:** The microcirculation is endowed with certain peculiar characteristics. First and foremost, the microcirculation is heterogenous with regard to rheologic and resistive properties in various organs and within the organ itself^4^. Heterogeneity of flow helps to supply adequate oxygen to tissues based on their metabolic demands. However, it also leads to microcirculatory units with unfavourable rheologic and/or resistive properties, making them weaker and thus more vulnerable to damage by hypoxia as encountered during sepsis. Secondly, in almost all vascular beds, there is a longitudinal and radial oxygen gradient such that the capillary pO_2_ and haemoglobin saturation are significantly lower than arterial values^5^. This results from oxygen unloading from arterial network to tissues, and the intrinsic oxygen consumption of vessel wall to sustain endothelial functions and vascular tone. These properties again make the exchange segment more prone to hypoxic damage. The microvascular haematocrit is lower than the systemic haematocrit, and is also heterogenously distributed^6^. This decrease is due to the Fahreus effect that induces axial migration of erythrocytes near the centre of vessels, resulting in differential erythrocyte and plasma velocities, and a dynamic decrease in intravascular haematocrit. The end result of all the above characteristics is a heterogeneity of blood flow and oxygen delivery in the microcirculation, resulting in vulnerable units prone to hypoxic damage.

**Importance of the microcirculatory endothelium:** The major cell types constituting the microcirculation include endothelial cells lining inside of the microvessels, smooth muscle cells present mostly in the arterioles, and components or blood i.e., erythrocytes, leucocytes, and plasma components. The endothelial cell surface in the microcirculation is the largest endothelial surface of the body - the largest ‘organ’ in the human body. The total endothelial surface area is approximately 4000 to 7000 m^2^ with most of the elements being within the microcirculation^7^.

By virtue of its anatomical location i.e., being a divide between the flowing blood within and the extra-cellular space beyond, the endothelium forms an interface between inflammation and coagulation^8^. It thus mediates and controls trans-endothelial exchanges between blood plasma and interstitial fluid, regulates the vasomotor tone by releasing vasodilating and vasoconstricting substances, maintains an anticoagulant state, and regulates transmigration of leukocytes into surrounding tissues.

The endothelium also plays a central role in regulation of microcirculatoiy perfusion^9^ by sensing flow, metabolic, and other regulating substances to alter arteriolar tone and capillary recruitment. Importantly, this endothelial sensing is capable of detecting downstream haemodynamic conditions e.g., lactate levels, and transmitting information upstream by cell to cell signaling, to adjust the perfusion accordingly.

## Pathophysiology of microcirculation in sepsis

The inflammatory mediators that herald sepsis, and the changes they induce in the macrohaemodynamics i.e., blood pressure, heart rate and oxygen extraction are well known. The ensuing section highlights the changes induced in the microcirculation by sepsis.

“Five to fifteen minutes after its (endotoxin) intravenous administration, there were strong waves of contraction along the small arteries, arterioles, and metaarterioles. These could arrest flow and last for several minutes. There Would afterwards be a phase of dilation, followed by a strong contraction. As time went on, the phases of relaxation became more prominent until preagonally there was a general and permanent vasodilation. The circulation would slow progressively until death.”

This early description^10^ of response of microvessels to endotoxin in guinea pig and mouse mesentery demonstrates the immediate arteriolar vasoconstriction response to endotoxin followed by the subsequent phases of changing microvascular tone and ultimate cardiovascular collapse.

The release of endotoxin or proinflammatory cytokines initiates a cascade of cellular and mediator changes in sepsis^11^. The corner stone of impaired homeostasis in sepsis is an inflamed microcirculation. It is clogged with microthrombi and leaks extensively and the central role in this microcirculatory dysfunction is in turn played by the endothelium^12^. It is damage to the endothelium that turns the usual water tight blood vessels into sieves allowing large amounts of protein rich fluid to leak into the subcutaneous tissues, causing extensive tissue oedema and intravenous dehydration. Activation of the coagulation cascade leading to intra-vascular thrombosis is also a result of the damaged endothelium that starts liberating progoabulant factors. Besides these alterations, the endothelium also fails to perform its regulatory functions, and its nitric oxide (NO) system is severely disturbed. There is a heterogenous expression of inducible nitric oxide synthase (iNOS) in the endothelium of different areas of organ beds. Areas that lack iNOS have less NO induced vasodilation and become underperfused resulting in pathological shunting of blood flow^13^,^14^.

The endothelium is not the only component of microcirculation to be altered. All other cellular components of the microcirculation also undergo deterioration during sepsis. Smooth muscle cells lining the arterioles loose their adrenergic sensitivity and tone^15^,^16^. The red blood cells become more rigid thus increasing the blood viscosity^17^. The percentage of activated neutrophils with decreased deformability and increased agreeability, due to upregulation of adhesion molecules also increases.

Recently, endothelial glycocalyx has also been shown to be involved in sepsis induced microcirculatory dysfunction^18^. The glycocalyx is a layer covering the endothelium and consists of endothelial cell derived proteoglycans, hyaluronan glycosaminoglycans, and selectively adsorbed plasma proteins^19^‐^21^. It is the first interface between blood and tissue, and is involved in physiological processes such as maintenance of vascular tone, mechanotransduction, and transport along vessels^20^,^21^. Its thickness regulates the organ blood flow and red blood cell velocity^22^‐^24^. It has been suggested that glycocalyx destruction occurs during endotoxemia, and this may participate in causing microvascular perfusion deficit^18^.

The aforesaid cellular alterations in the microcirculation lead to impairment of all three functional elements of the microvascular network. The arterioles are hyporesponsive to vasoconstrictors and vasodilators despite the elevated levels of catecholamines^25^, perfused capillaries are reduced in number, and venules are obstructed by the sequestered neutrophils. In the capillaries, besides a decreased density, there also occurs increased heterogeneity and an increase in the proportion of stopped and intermittently perfused capillaries^26^–^30^. This shut-down of the vulnerable microcirculatory units in the organ beds promotes the shunting of blood and hence oxygen, from arterial to venous compartment leaving the microcirculation hypoxic, along with a decrease in oxygen extraction. The local microcirculatory partial pressure of oxygen drops below the venous oxygen pressure. This dillerence has been termed the “pO_2_ gap” and is an indicator of the severity of functional shunting^9^. The systemic manifestation of this pathologic shunting is seen as a deficit of oxygen extraction by tissues with an apparently normal delivery, and raised venous pO_2_, lactate, and gastric CO_2_ levels. In addition, the blood flow regulation of microcirculation is severely disrupted.

## Microcirculatory perfusion as an endpoint

Much of the research pertaining to resuscitation during sepsis has focused on restoring the macrodynamics of circulation such as blood pressure, oxygen delivery and oxygen extraction ratio. The pathologic shunting occurring in the microcirculation is not depicted by systemic haemodynamic derived and oxygen derived variables. The difference between macrocirculation and microcirculation was recognized very early on^10^ when it was pointed that changes in total peripheral resistance could not provide information regarding local vascular resistance changes since “dilation in one vascular bed may be accompanied by constriction elsewhere”. Also, the cause of alterations in the macrohaemodynamics lies in the microcirculation e.g., the decrease in systemic vascular resistance and hypotension result from arteriolar vasodilatation and hypovolemia from capillary leak. Thus, it needs to be answered whether resuscitating the microcirculation rather than the macrocirculation will finally answer the quest for improving survival in sepsis.

There is previous evidence that resuscitating the macrohaemodynamics is not always associated with improved microhaemodynamics, organ function, or survival^31^–^35^. A study by LeDoux and colleagues^35^ observed the effect of norepinephrine on global haemodynamic parameters and measures of tissue oxygenation during septic shock. While the mean blood pressure increased from 65 to 85 mmHg along with expected increase in heart rate and cardiac index (*p*<0.05), there was no improvement in organ function or tissue oxygenation as evidenced by decrease in urine output, no change in capillary red blood cell velocity, fall in capillary blood flow and increase in gastric pCO_2_. The authors thus concluded that resuscitation of mean blood pressure or cardiac output alone in septic shock is inadequate. Microcirculatory independence from arterial blood pressure in septic shock has also been proven using direct imaging of microcirculation^33^,^34^. DeBacker et al^33^ reported a significant decrease in vessel density and proportion of small perfused vessels in septic patients, the alterations being more severe in non-survivors and were not related to the mean arterial pressure. Sakr and colleagues^34^ further explored these findings by studying the microcirculation in 49 septic patients. The small vessel perfusion was seen to improve rapidly in survivors as compared to non-survivors, with no difference in the global haemodynamic variables. Together with the evidence showing that organ function improves and mortality decreases when resuscitation boosts microcirculatory flow^36^, the microcirculation does appear to be a new target for resuscitation during sepsis^6^.

## Therapies for optimizing microcirculation

Even though several experimental data are available regarding effect of various therapeutic interventions on microcirculation, human data is still limited^37^. The ideal modality to resuscitate the microcirculation and the endpoints to be achieved still remain to be defined. Against this background, the following section explores the suggested modalities for microcirculatory therapy.

The commonly practiced combination therapy developed by Rivers and colleagues^38^ in their protocol of “early goal directed therapy” involves achieving macrohaemodynamic end-points i.e., central venous pressure of 8-12 mmHg, addition of vasopressor to maintain mean arterial pressure ≥65 mmHg, measurement of central venous oxygen saturation, red cell transfusion, and/or inotropic agents to increase central venous oxygen saturation to 70%. It has also however, been shown to improve the microcirculatory flow, organ function and ultimately the survival.

*Intravascular resuscitation*. Both crystalloid and colloid infusions recruit vessels, and improve barrier function and oxygen transport in the microcirculation^39^–^43^. Although prospective studies regarding the choice of fluid for resuscitation in patients with septic shock are lacking, a large prospective, controlled, randomized, double-blind study comparing 4 percent human albumin solution with 0.9 percent sodium chloride in critically ill patients requiring fluid resuscitation (the Saline vs. Albumin Fluid Evaluation (SAFE) study) has recently been published^44^. The results of this study show identical mortality rate in patients receiving albumin or 0.9 percent sodium chloride. However, subgroup analysis reveals that albumin might have some (albeit not statistically significant) benefit in patients with severe sepsis.

Blood is a better oxygen carrier and hence improves oxygen delivery to microcirculation more than with either crystalloid or colloid. Certain data however suggests that erythrocyte transfusion may not improve the microcirculatory perfusion due to the 2-3-DPG depletion, poor erythrocyte deformability, and erythrocyte interaction with endothelium and other blood cells^6^. Given the variable effects of erythrocyte transfusion it is emphasized that use of erythrocyte transfusion needs to be analyzed according to the baseline haematocrit, while also keeping in mind the storage time and presence or absence of residual leukocytes in transfused products.

*Nitric Oxide Synthase (NOS) inhibitors*. The concept of NO inhibition therapy for sepsis is debatable at present^45^, with the role of NO itself being equivocal with respect to its effect on microcirculation^46^. Improvement in microvascular blood flow has been shown with both, NO donors^47^, and iNOS inhibitors^48^,^49^.

In sepsis, overproduction of NO from endothelial cells through the upregulation of iNOS has been associated with impaired vascular reactivity, capillary leak, erythrocyte deformiability and refractory hypotension^50^. This is also known to inhibit mitochondrial respiration, reversibly or irreversibly, depending on the duration of NO exposure and the mitochondrial complex inhibited^51^–^54^.

Early data in septic shock patients treated with NOS inhibitors showed increasing blood pressure and decreasing dose of vasopressors^55^. However, a subsequent randomized controlled multicenter phase III trial had to he stopped when interim analysis showed increased mortality with the NO synthase inhibitor 546C88^56^. Other authors have also noted raised mortality despite an improvement in the general haemodynamic parameters with usage of NOS inhibitors^57^,^58^.

Certain authors also suggest that completely inhibiting vasodilation is not the appropriate answer to sepsis. A more specific approach by inhibiting the inducible form of NOS has been studied. Following the application of 1400W (a synthetic blocker of inducible NOS) in a pig endotoxemia model, microvascular perfusion was restored by a redistribution within the gut wall and/or an amelioration of the cellular respiration^59^.

A new perspective in the debate regarding the role of iNOS/NO in septic shock has recently been put forward by the study of Bateman and colleagues^45^. The authors noted that timing as well as degree of iNOS/NO inhibition may be an important determinant in altering prognosis in septic shock. They found increased oxygen consumption by inhibiting iNOS/NO overproduction at the onset of hypotensive sepsis. In contrast to earlier trials, the therapy was initiated very early in sepsis and no attempt was made to normalize the mean arterial pressure, rather the aim was to maintain the NO level at baseline value.

In recent animal studies it has been observed that combination of fluid therapy with iNOS inhibition was successful in recruiting vulnerable microcirculation in the intestine, while fluid therapy alone was unable to do so^41^,^59^.

*Steroids*. Use of steroids in sepsis represents a non-specific approach towards modulation of the systemic inflammatory response, and inhibition of iNOS. However, this is a time dependent phenomenon since sepsis evokes NO induced inhibition of glucocorticoid receptor. Following the recent large, European multicenter trial^60^ which failed to show any mortality benefit with steroids in septic shock, never recommendations^61^ suggest that only adult septic shock patients in whom blood pressure is poorly responsive to fluid resuscitation and vasopressor therapy should receive steroid therapy. For improvement of autoregulation of microcirculation, relatively higher doses are required and thus not recommended for clinical use in sepsis. These recommendations have dampened the earlier enthusiasm created by the study of Annane et al^62^ regarding the use of steroids in septic shock. The authors had found adrenal insufficiency in greater than 50% patients of septic shock and these patients had responded well to low dose hydrocortisone therapy.

*Statins*. The role of statins in sepsis has been reviewed in great detail elsewhere^63^. Statins are widely used as cholesterol-lowering agents but appear to have an anti-inflammatory action during sepsis. The primary mechanism of their action in sepsis is by increasing expression of eNOS (endothelial nitric oxide synthase – constitutive enzyme), along with a down-regulation of iNOS. Together, this increases NO levels, restoring the endothelial functions. Other beneficial effects of statins in sepsis may also include its antioxidant activity and alterations in development of vascular atherosclerosis^63^. The future promise of statins in sepsis is a subject of great interest and current research^64^,^65^.

*Vasodilators*. As per the shunting theory of sepsis, correction of the condition should occur by recruitment of the shunted microcirculatory units. Applying strategies to ‘open the microcirculation’ by vasodilation is thus expected to promote microcirculatory flow by increasing the driving pressure at the entrance of the microcirculation and/or decreasing the capillary afterload^66^. In the very early stages of sepsis although eNOS decreases causing impaired endothelium-dependent vasodilation, the iNOS release contributing to hypotension may take several hours^67^. Thus, an early administration of a NO donor may be beneficial to preserve tissue perfusion. In a recent trial by Assadi et al^68^ the use of sodium nitroprusside (SNP), a NO donor, during early severe sepsis was observed to improve the hepatosplanchnic microcirculatoiy blood flow. Recruitment of microcirculation by vasodilator therapy in the form of NO donors^41^, nitroglycerin^47^, prostacyclin^69^ and even topical acetylcholine^33^ has been found to be effective for microcirculatory recruitment. Pending However, is the usefulness of these approaches in clinical course^37^.

*Vasopressors/Inotropes*. Commonly recommended vasopressors/inotropes in sepsis include dopamine, norepinephrine, epinephrine and dobutamine. While these are potent for correcting systemic haemodynamics, their use should be viewed with caution for intent of improving microcirculation. Their detrimental effects on regional perfusion are well described^70^–^72^. Dobutamine can improve but not fully reverse microcirculatoiy alterations in patients with septic shock^73^. Vasopressin, a more recently investigated vasopressor in sepsis, has been shown to increase urine output while raising the blood pressure^74^,^75^, but it has also been seen to cause microcirculatory shutdown^76^. It appears that further studies are required to determine the best vasopressor for microcirculatory septic shock^36^.

*Combination therapy*. Combination of fluid therapy with vasoactive and inotropic support is effective in restoring the microcirculation^77^. Non-responders to this therapy have a poor prognosis. A seemingly contradictory combination of NO donor and iNOS inhibitor may also prove to be successful in recruitment of microcirculation^77^.

*Activated Protein C (APC)*. Protein C, a component of the natural anticoagulation system, is an antithrombotic serine protease that is activated to APC in the body by thrombin thrombomodulin complex. Deficiency of APC has been shown to increase morbidity and mortality in patients of sepsis and septic shock^78^,^79^. Therapy with APC aims directly at the pivot of sepsis, the endothelium, by a multimodal mechanism possessing anti-inflammatory properties independent of its anti-coagulation properties. It inhibits iNOS expression^80^, decreases level of TNF α^81^, reduces leucocyte activation and release of reactive oxygen species, improves capillary density^82^, and acts on coagulatory pathways^83^ by inhibiting factors Va and VIIIa, as well as by promoting fibrinolysis. The only adverse effect to be considered was the risk of bleeding. Despite the encouraging report of successful use of APC^84^, the most recent guidelines however, limit its use only to very sick patients of sepsis^61^. Lehmann et al^85^ have published a very elegant study regarding the effect of APC on the microcirculation and cytokine release, during experimental endotoxemia in rats. The authors observed APC to attenuate deterioration of microvascular blood flow by decreasing leucocyte adherence, plasma extravasation and a decrease in systemic cytokine IL-1β. These findings are consistent with those of earlier trials regarding, effect of APC on microcirculation^82^,^86^–^87^. APC also decreases the oxidative stress and glycocalyx destruction during endotoxemia^18^.

*Other pharmacologic interventions*. Arachidonic acid metabolites are powerful lipid mediators playing a key role in microcirculatory failure. They increase interleukin-1 release by macrophages in sepsis. It has been demonstrated that pharmacologic inhibition of leukotrienes^88^,^89^ and thromboxane A2^90^, and usage of thromboxane receptor antagonists is beneficial during sepsis. On the other hand, prostaglandin El infusion for 7 days improved survival and decreased organ failure in patients of ARDS^91^.

Preliminary animal data has shown benefits of cholinesterase inhibition with physostigmine or neostigmine in survival during sepsis^92^. The probable mechanism of action is the activation of the cholinergic anti-inflammatory pathway^93^. However, there is no data regarding its effect on the microcirculation as yet.

Levosimendan is a never vasoactive drug that acts by Ca^+2^ sensitization in the myocardium and the opening of the K_ATP_ channels in vascular smooth muscle cells. It has been shown to improve the cardiac dysfunction of sepsis at the “macro” level, and also improve the tissue pO_2_ without much alterations in the microcirculation^94^.

## Assessment of microcirculation

Till date, there is no single objective gold standard to assess the microcirculation. In clinical practice, microcirculatory perfusion has been traditionally judged by the color, capillary refill and temperature of the distal parts of the body (i.e., finger, toes, earlobes and nose). Amongst the investigational modalities available to assess microcirculation, both indirect indicators as well as direct techniques exist^6^, even though any single objective reliable method is still not recognized. Indirect techniques involve measurement of ‘downstream’ global derivatives of microcirculatory dysfunction such as lactate, carbondioxide, and oxygen saturation. The direct imaging of microcirculatory perfusion seems a superior approach to assessment of microcirculation. Invention of microscope is perhaps the single most important advancement in technology linked to discovering the microcirculation, since experimental investigation of the microcirculation began soon after its advent. Studies of human microcirculation began at the end of 19^th^ century, with Hueter using a microscope with reflected light to investigate vessels on inner border of lower lip.

## Indirect assessment of microcirculation:

*Lactate levels* in the blood are thought to reflect anaerobic metabolism associated with tissue dysoxia and hence may predict the prognosis and response to therapy. However, the balance between lactate production due to global (shock, hypoxia), local (tissue ischemia), and cellular (mitochondrial dysfunction) factors on the one hand, and lactate clearance depending on metabolic liver function on the other hand, make the interpretation of lactate levels uncertain and difficult^95^. Recent evidence also suggests that blood lactate concentration may be affected by other factors such as altered pyruvate dehydrogenase, Na+, K+-ATPase activity and increased glycolysis rate. Even so, increased lactate levels do help to identify patients with tissue hypoperfusion, and if levels are markedly elevated, serve as a trigger for initiating early goal directed therapy^61^. The current recommendations^96^ advocate use of serum lactate levels to identify patients with “crytic shock” i.e., preserved macrohaemodynamics with altered microcirculation.

*Mixed venous oxygen saturation (SvO2)* can be measured using a pulmonary artery catheter and is thought to reflect the average oxygen saturation of all perlused microvascular beds. But in sepsis, microcirculatory shunting can cause normal SvO2 despite existence of severe local tissue dysoxia^9^. Even though maintaining SvO2>65% is advocated as a recommendation to treat severe sepsis and septic shock, it may not reflect restoration of local tissue oxygenations^97^.

An appealing alternative to the evaluation of tissue dysoxia is the use *tonometry* of gastrointestinal tract. Tonometry is based on the principle that during hypoxia, anaerobic metabolism leads to production of acids which are buffered by bicarbonate ions leading to increased carbondioxide tension in tissues. The optimal site for monitoring tissue pCO_2_ is unclear^37^. Intestinal, gastric, oesophageal and rectal pCO_2_ have all been investigated. Recently, sublingual mucosa and skin, which are not a part of splanchnic circulation have been investigated and appear promising. Sublingual capnometry has numerous advantages over gastric tonometry. It is simple to perform, non-invasive, produces immediate result, and can be used at the bedside. It does not require premedication and acid suppression therapy, and patients do not have to be withheld from enteral feeding. The earlier index of measuring tissue dysoxia by tonometry was pHi wherein a value of <7.32 indicated ischaemia. However, measurement of the difference between tissue (intestinal) pCO_2_ and arterial pCO_2_ has been found to be a better indicator since the arterial pCO_2_ fluctuates in ventilated patients. In the stomach, normal gastric-arterial pCO_2_ gradient is <7 mmHg. Sublingual pCO_2_ values have been found to correlate well with gastric intramucosal pCO_2_ values^98^. The baseline difference between sublingual pCO_2_ and arterial pCO_2_ values is a better predictor of survival than the change in lactate or SvO2^99^.

## Direct assessment of microcirculation:

*Intravital microscopy (IVM)* depends on transor epi-illumination and thus observations are limited to superficial layers of thin tissues only. By using fluorescent dyes a higher contrast is possible as well as specific cells can be labeled for visualization and quantification. Its use has been primarily limited to animal studies because of the potentially toxic effects of dyes, and the limited access of tissues allowed with its usage. Its use in humans is usually restricted to the eye, skin and the nail fold.

*Laser Doppler* involves the principle of detection of frequency shift in laser light alter it encounters flowing erythrocytes. It measures the velocity of microcirculatory flow in a small area of microcirculation, being an average of the velocities in all the vessels present in the measured volume. It can be used to measure the flow in skin, muscle, gastric mucosa, rectum and vagina. It has been validated in experimental models and gives an accurate assessment of changes in velocity induced by pharmacologic interventions. The limitations of Laser Doppler include the estimation of an average flow in aboutonly about 1 mm^3^ of tissue, disregard of the morphology of microvessels, the direction of flow, and heterogeneity of blood flow in the microcirculation, as well as failure to account for any changes in haematocrit.

*The scanning Laser Doppler* technique is an advancement over the conventional technique that allows two dimensional visualization of the microcirculation. It has been used to assess perfusion for oesophageal or colonic anastomosis, and cutaneous perfusion of the loot during arterial cannulation in critically ill patients.

*Orthogonal Polarization Spectral (OPS) lmaging* is a newer noninvasive method for direct visualization of microcirculation using green polarized light to illuminate the area of study^100^. The polarized light is scattered by the tissue and collected by an objective lens. A polarization filter or analyzer oriented orthogonal to the polarized light is placed in front of the imaging camera. This analyzer eliminates the reflected light which is scattered at or near the surface of the tissue, while depolarized light scattered deeper within the tissue passes through the analyzer. When this depolarized light coming from deeper tissues passes through absorbing structures close to the surface, such as blood vessels, high contrast images of nlicrocirculation are formed. It is especially useful for studying the tissues protected by a thin epithelial layer, such as mucosal surfaces. Incorporated in a hand held type of microscope, OPS imaging was introduced clinically to first identify pathologies during surgery. The sublingual area is the most frequently investigated mucosal surface. That the sublingual site indeed represents microcirculation of other areas finds favour with certain authors^37^.

Limitations of OPS imaging in sublingual region include movement artifacts such as respiration, and presence of various secretions such as blood and saliva. Also, patients have to be cooperative or adequately sedated such that they do not bite the device. The technique can investigate only those tissues that are covered with a thin epithelial layer, and of course internal organs are not available except during intraoperative conditions. It does not give the exact measurement of red blood cell flow velocity in individual vessels. What it does allow, is prediction of a semiquantitative flow score based on average score over a maximum of 12 quadrants (three regions X four quadrants per region), derived from the overall flow impression of all vessels with a particular range of diameter in a given quadrant.

The flow score is a semi-quantitative one, and whether the flow score from 0 to 3^101^ is actually a linear relationship with the actual flow is also not established. With repeated measures, selecting the exact site as before is also a difficult task. An improvement in the OPS imaging is the sidestream dark-field (SDF) imaging. It consists of a light guide surrounded by 530 nm light-emitting diodes, a wavelength of light that is absorbed by haemoglobin of erythrocytes, allowing their observation as dark cells flowing in the microcirculation. As compared to OPS it offers the advantage of improved image quality, relative technical simplicity, and lack of need of a high-powered light source^94^.

## Future aspects

With several clinical and laboratory indicators of identifying hypoperf ision due to the microcirculation dysfunction being available, it is perhaps time to recognize shock in sepsis keeping tissue hypoperfusion as distinct from hypotension. A perfusion based scoring system has been proposed by Spronk et al^97^. It emphasizes the need of extending recognition of shock severity to include microcirculatory parameters, besides global haemodynamic and oxygen-derived parameters.

Therapy in shock should be aimed at optimizing cardiac function, arterial hemoglobin saturation, and tissue perfusion. This not only includes correction of hypovolemia, but the restoration of an evenly distributed microcirculatory flow and adequate oxygen transport as well. The role of vasodilators in recruiting the microcirculation will need to be looked into further.

Direct monitoring of sublingual microcirculation monitoring appears to be a promising endpoint for resuscitating the microcirculation. An integrative approach incorporating both macrocirculatory and microcirculatory haemodynamic data may indeed hold the answer to resuscitation in sepsis.
